# Supplementary material for: Preparation of Boron/Sulfur-Codoped Porous Carbon Derived from Biological Wastes and Its Application in a Supercapacitor
Source: Nanomaterials (Basel). 2022 Apr 1;12(7):1182. doi: 10.3390/nano12071182 (PMC9000563; doi:10.3390/nano12071182)
Supplement: Supplementary file 1 [file nanomaterials-12-01182-s001.zip › nanomaterials-1642079-supplementary.pdf]

nanomaterials

MDPI

*Supplementary Materials*

# Preparation of Boron/Sulfur-Codoped Porous Carbon Derived from Biological Wastes and Its Application in a Supercapacitor

**Yanbin Wang [1,2,3], Dian Wang [1,2,3], Zhaoxia Li [1,2,3,*], Qiong Su [1,2,3,*], Shuai Wei [1,2,3], Shaofeng Pang [1,2,3], Xiangfei Zhao [1,2,3], Lichun Liang [1,2,3], Lihui Kang [1,2,3] and Shijun Cao [1,2,3]**

[1] School of Chemical Engineering, Northwest Minzu University, Lanzhou 730030, China; ybwang@126.com (Y.W.); d_wang1997@163.com (D.W.); w17803870317@163.com (S.W.); pangshaofeng2006@163.com (S.P.); woaini6704259@163.com (X.Z.); fgl770880llcfgl@163.com (L.L.); lihuikang0325@163.com (L.K.); a2848695227@163.com (S.C.)

[2] Key Laboratory of Environmentally Friendly Composite Materials of the State Ethnic Affairs Commission, Key Laboratory of Utility of Environmental Friendly Composite Materials and Biomass in Universities of Gansu Province, Lanzhou 730030, China

[3] Engineering Research Center of Biomass-Functional Composite Materials of Gansu Province, Lanzhou 730030, China

* Correspondence: zhaoxia_90dong@163.com (Z.L.); hgsq@xbmu.edu.cn (Q.S.)

## 1.1. Material Characterization

Scanning electron microscopy (SEM, SU8000) and transmission electron microscopy (TEM, FEI Tecnai G2 F20) were employed to analyze the morphologies and microstructures of samples. The nitrogen adsorption and desorption isotherms at 77 K (Micromeritics ASAP) were used to examine the porosities of samples. The specific surface area was calculated via the Brunauer–Emmett–Teller (BET) model. X-ray diffraction (XRD) was recorded on a diffractometer (X'Pert PRO) at 40 kV and 40 mA in the scanning range of 2θ = 10–80° with a speed of 10°/minute. Raman spectra were collected under 532 nm laser excitation at room temperature (LabRAM ARAMIS). Surface elements of samples were studied by X-ray photoelectron spectroscopy (T XPS, VG ES-CALAB 210) with an exciting source of Al Ka. The wettability of the sample were measured by static water contact angle at ambient temperature through a contact angle goniometer (HARKE-SPCAX3).

## 1.2. Electrochemical Measurements

The preparation of the working electrodes included the following steps: first, the B/S-$SC_S$–1, PVDF, and conductive acetylene black with 8:1:1 wt% ratios were mixed uniformly, and then a few drops of NMP was added to prepare a uniform slurry. After being ground, the mixture was uniformly coated onto nickel foam (Three-electrode: (1 × 1 $cm^2$ ); two-electrode:( For the 2 cm in diameter) and dried at 60°C. Finally, the nickel foam was pressed with a hydraulic device for 1 min at 10 MPa. The mass loading of working electrode is ~3 mg in three-electrode system, The mass loading of working electrode is 6 mg in two-electrode system. Three-electrode system tests were carried out in 6 M KOH aqueous solution with a Hg/HgO electrode as the reference electrode and a slice of platinum as the counter electrode. The electrochemical performance of working electrodes, such as the cyclic voltammetry (CV), galvanostatic charge/discharge (GCD), and electrochemical impedance spectroscopy (EIS), was investigated on a CHI760E electrochemical workstation (Chenhua Instruments Co., Ltd., Shanghai, China).

In the three-electrode configuration, the positive, negative and separator were assembled in a small split test cell (EQ-STC, Hefei Kejing Material Technology Co., Ltd). The value of the specific capacity was calculated by GCD as follows [58]: $C = I\Delta t/m\Delta V$, where C(F $g^{-1}$) represents the specific capacitance, I(A) is the discharge current, Δt (s) is the discharge time, m(g) is the mass of the electrode, and ΔV(V) is the potential window. In the two-electrode configuration, the value of the specific capacity was calculated by GCD as follows [60]: $C = 4I\Delta t/m\Delta V$, where C(F $g^{-1}$) represents the specific capacitance, I(A) is the

discharge current, Δt(s) is the discharge time, m(g) contains the mass of active species from both positive and negative electrodes, and ΔV(V) is the potential window. The energy density and power capability were calculated by the following equation [62]: $E = C\Delta V^2/8\times3.6$ and $P = E/\Delta t$, where (E, W h kg$^{-1}$) is the energy density, (E, W h kg$^{-1}$) is the power density, C(F g$^{-1}$) means the specific capacitance of the supercapacitors, ΔV(V) is the potential window, and Δt (s) is the discharge time.

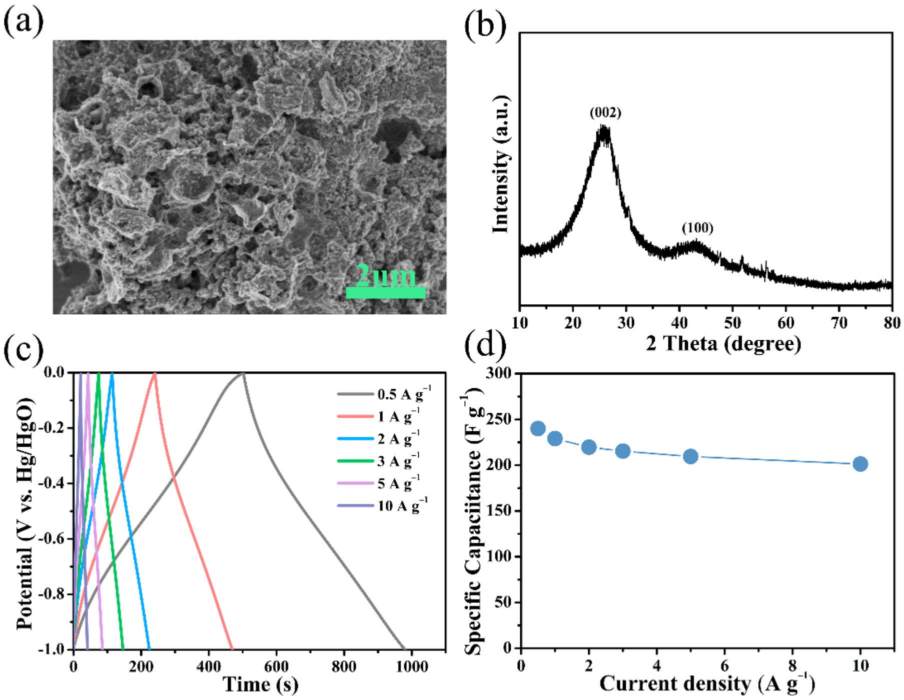


**Figure S1.** (**a**) SEM image and (**b**) XRD pattern of walnut peel derived porous carbon; (**c**) GCD curves in three-electrode system and (**d**) Specific capacitances at different current densities.

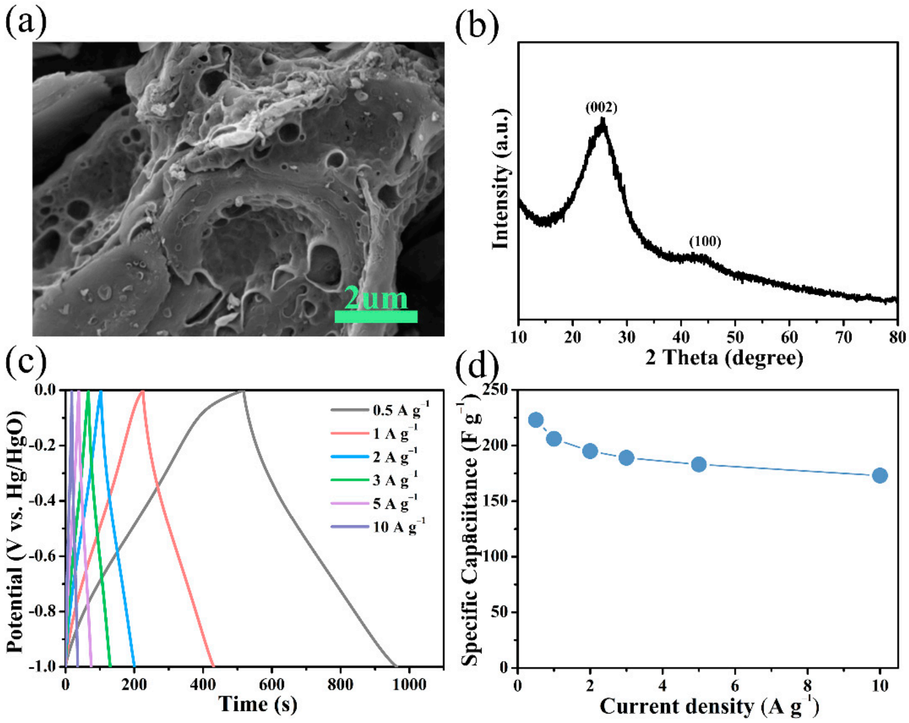


**Figure S2.** (**a**) SEM image and (**b**) XRD pattern of wheat straw derived porous carbon; (**c**) GCD curves in three-electrode system and (**d**) Specific capacitances at different current densities.

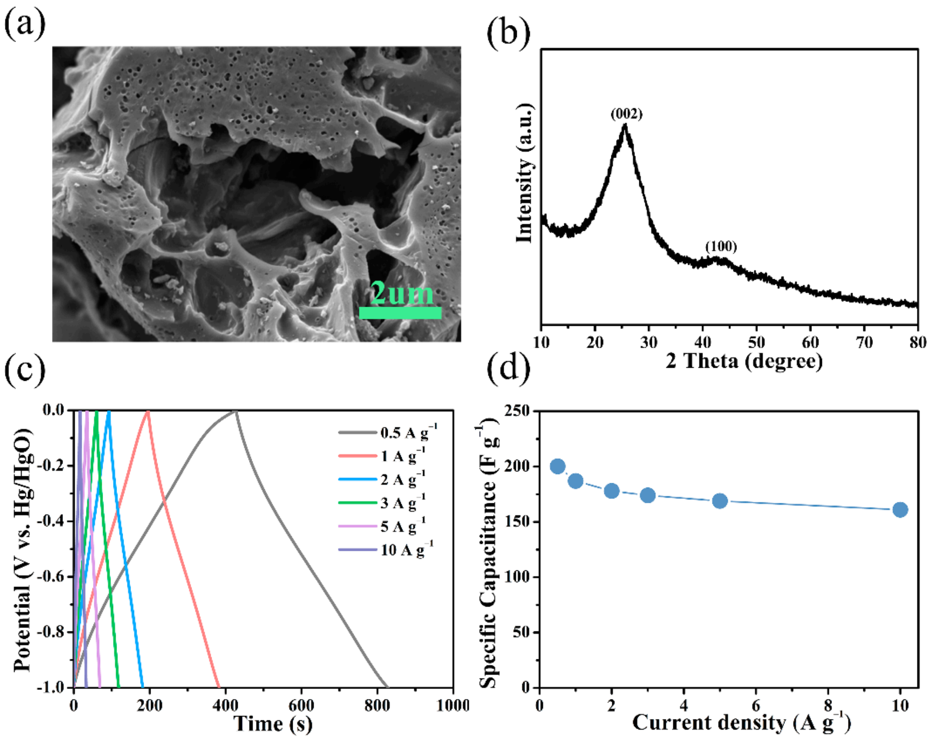


**Figure S3.** (**a**) SEM image and (**b**) XRD pattern of corn stalks derived porous carbon; (**c**) GCD curves in three-electrode system and (**d**) Specific capacitances at different current densities.

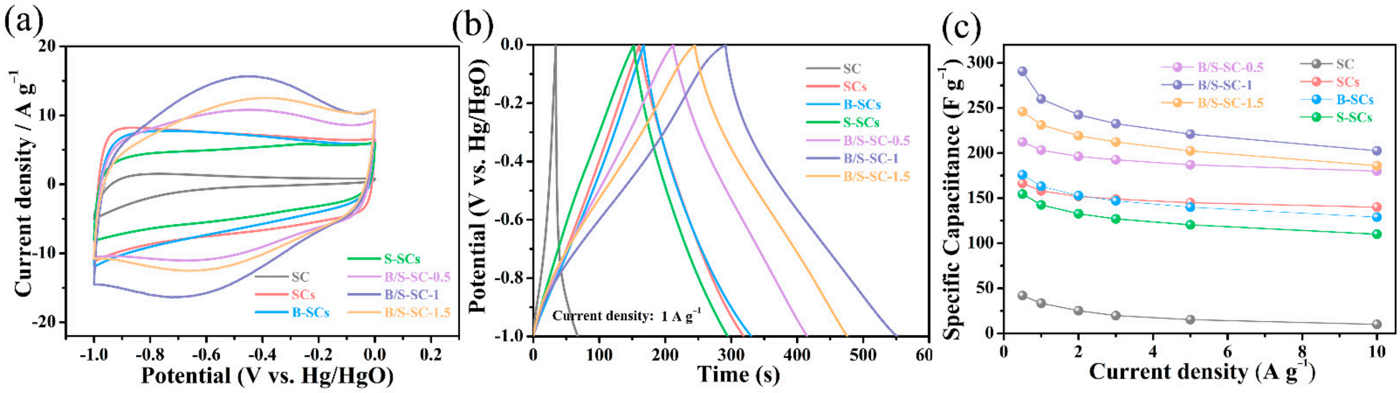


**Figure S4.** (**a**) CV curves at 50 mV $s^{-1}$ scan rate for electrodes containing all samples as active ma-te-rials; (**b**) GCD curves at 1 A $g^{-1}$ scan rate for electrodes containing all samples as active materi-als; (**c**) Ratio capacitances of all samples under diverse ampere densities.

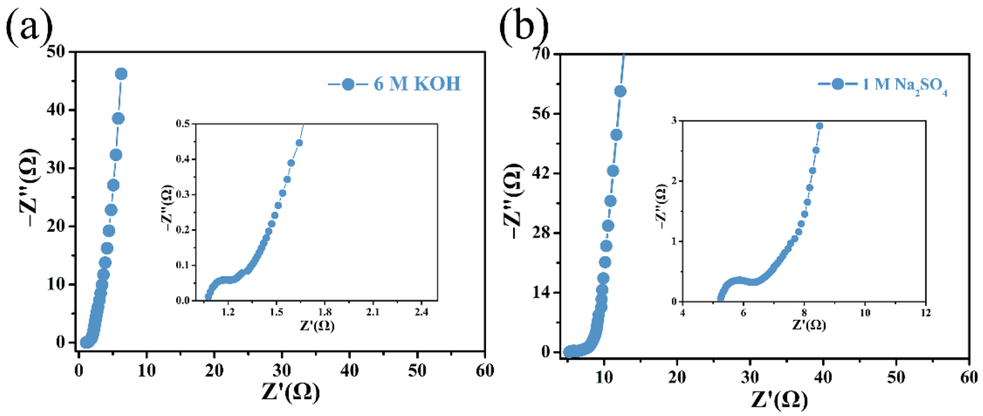


**Figure S5.** (**a**) Nyquist plots of B/S-SCs-1// B/S-SCs-1 symmetric supercapacitor using 6 M KOH electrolyte, (**b**) Nyquist plots of B/S-SCs-1// B/S-SCs-1 symmetric supercapacitor using 1 M $Na_2SO_4$ electrolyte.

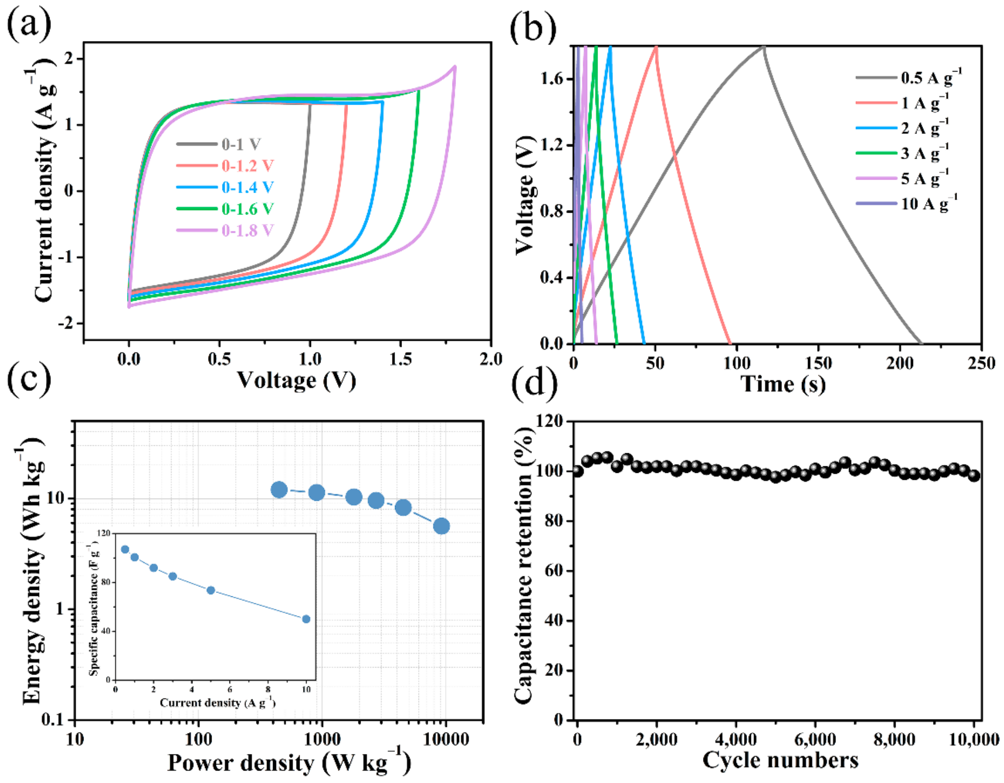


**Figure S6.** Electrochemical performance of walnut peel derived porous carbon symmetric supercapacitor using 1 M $Na_2SO_4$ electrolyte. (**a**) CV curves obtained varying voltage from 1 to 1.8 V at scan rate of 50 mV $s^{-1}$, (**b**) GCD curves at various current densities from 0.5 to 10 A $g^{-1}$, (**c**) The specific capacitances at different current densities and Ragone plots, (**d**) Cycling stability and coulombic efficiency of the device at 10 A $g^{-1}$.

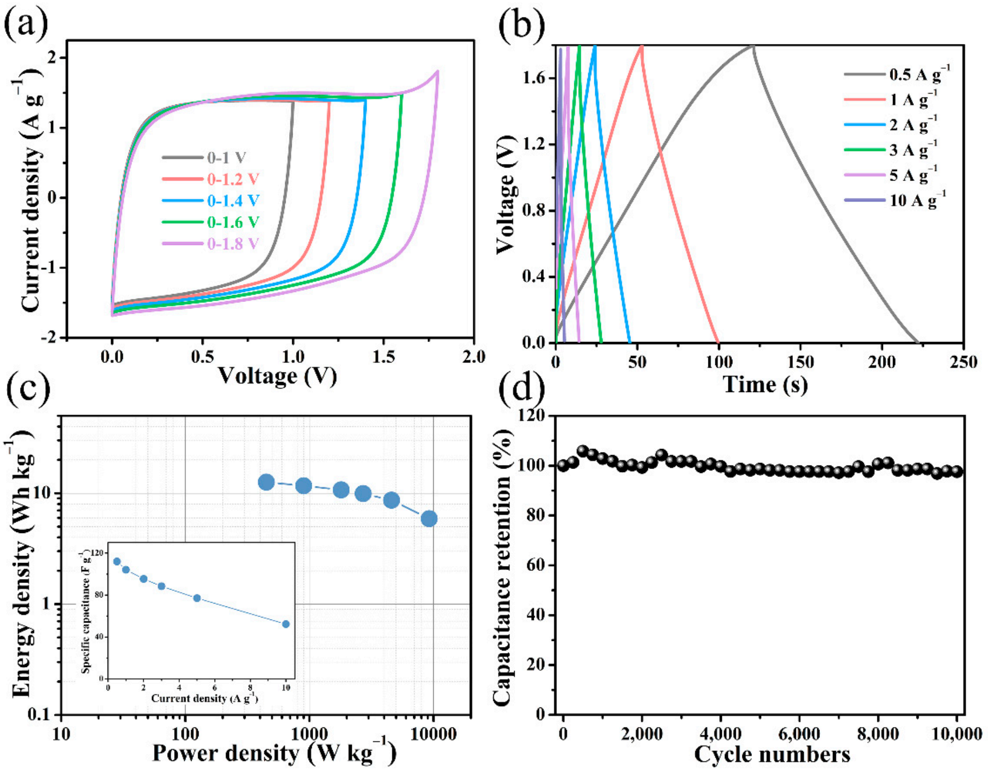


**Figure S7.** Electrochemical performance of wheat straw derived porous carbon symmetric supercapacitor using 1 M $Na_2SO_4$ electrolyte. (**a**) CV curves obtained varying voltage from 1 to 1.8 V at scan rate of 50 mV $s^{-1}$, (**b**) GCD curves at various current densities from 0.5 to 10 A $g^{-1}$, (**c**) The specific capac-itances at different current densities and Ragone plots, (**d**) Cycling stability and coulombic efficiency of the device at 10 A $g^{-1}$.

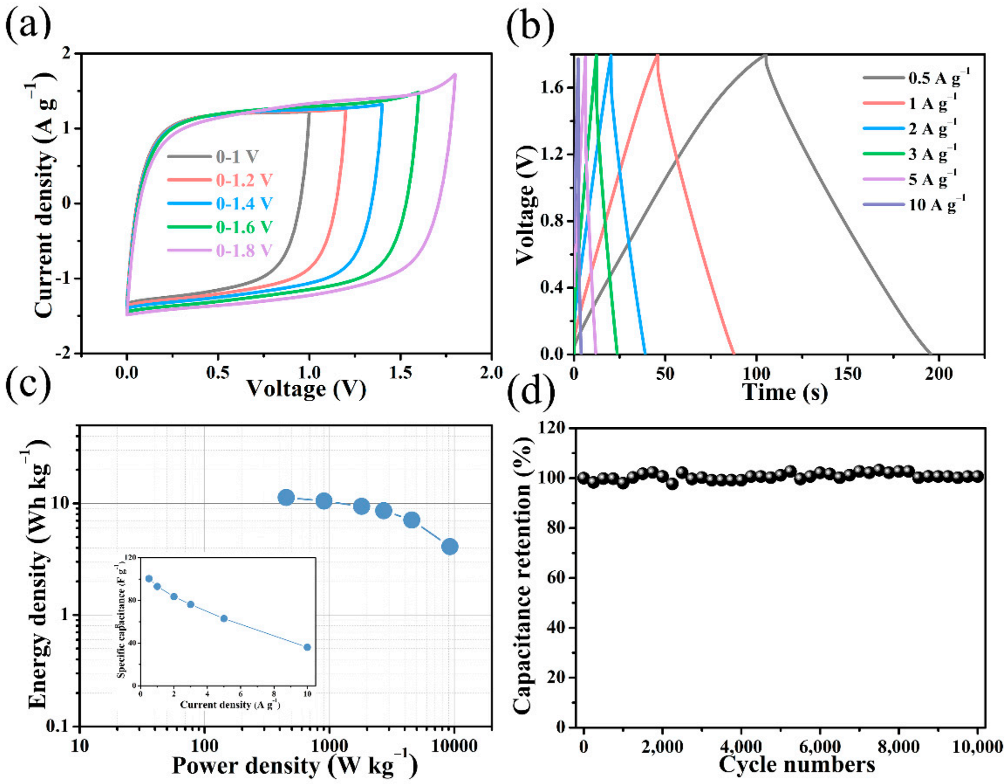


**Figure S8.** Electrochemical performance of corn stalks derived porous carbon symmetric supercapacitor using 1 M $Na_2SO_4$ electrolyte. (**a**) CV curves obtained varying voltage from 1 to 1.8 V at scan rate of 50 mV $s^{-1}$, (**b**) GCD curves at various current densities from 0.5 to 10 A $g^{-1}$, (**c**) The specific capacitances at different current densities and Ragone plots, (**d**) Cycling stability and coulombic efficiency of the device at 10 A $g^{-1}$.

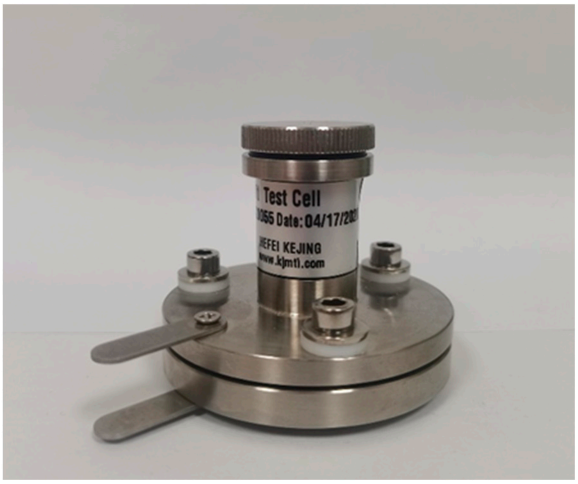


**Figure 9.** The real image of the symmetric device.

## References


58. Yang, W.; Yang, W.; Kong, L.; Song, A.; Qin, X.; Shao, G. Phosphorus-doped 3D hierarchical porous carbon for high-performance supercapacitors: A balanced strategy for pore structure and chemical composition. *Carbon* **2018**, *127*, 557–567.
60. Guan, L.; Pan, L.; Peng, T.; Gao, C.; Zhao, W.; Yang, Z.; Hu, H.; Wu, M. Synthesis of Biomass-Derived Nitrogen-Doped Porous Carbon Nanosheests for High-Performance Supercapacitors. *ACS Sustain. Chem. Eng.* **2019**, *7*, 8405–8412.
